# Trends and hotspots on hymenoptera venom immunotherapy: a bibliometric and visualized analysis of research from 2014 to 2024

**DOI:** 10.3389/fimmu.2025.1546704

**Published:** 2025-04-30

**Authors:** Yun Luo, Minxia Guan, Yichuan Yu

**Affiliations:** Emergency Department, The Affiliated Yongchuan Hospital of Chongqing Medical University, Chongqing, China

**Keywords:** bibliometric analysis, venom immunotherapy, CiteSpace, allergy, hymenoptera

## Abstract

**Objective:**

Recently, the application of hymenoptera venom immunotherapy (VIT) has been extensively studied in the medical community. Nevertheless, there are still very few bibliometric analyses devoted to this field. Therefore, this study aims to provide a comprehensive overview of the development of research in the past 11 years and clarify future research directions and trends.

**Methods:**

From 2014 to 2024, articles related to “hymenoptera venom immunotherapy “and “Allergy” were from the core collection of Web of Science. This visual analysis included examining annual productivity, cooperation between countries and institutions, co-cited references, author and journal cooperation networks, keyword co - occurrence, and their respective clustering and trends.

**Results:**

A total of 332 articles on the allergy caused by hymenoptera VIT were included in this study. Germany, Azienda Ospedaliera Universitaria Integrata Verona and Golden, David B K are the most productive countries and institutions respectively. Analysis of the top 10 literatures with co-citation frequency found that 4 were expert consensus and guidelines, 4 were single-center (or multi-center), open, randomized controlled trials, and 2 were systematic reviews. Keyword cluster analysis showed that wasps were identified as the primary focus of hymenoptera VIT in the past, and mastocytosis and hymenopteran venom allergy were the current research hotspots.

**Conclusion:**

Recent studies on hymenoptera VIT have shown that today’s VIT increasingly emphasizes individualized and refined treatment. However, there is a lack of evidence of multicenter randomized controlled trials (RCTs) in this field. Further investigation is warranted to bridge this research gap.

## 1 Introduction

Venom immunotherapy (VIT) is the single best and only disease-modifying treatment mode for the treatment of hymenoptera venom allergy (HVA), affecting up to 3% of adults and 0.8% of children Settipane et al. (1) Floyd et al. (2) The first report of specific immunotherapy (IT) for HVA was considered to be Braun’s use of bee venom to treat bee allergy patients in 1925 [3]. Although Mary Hewitt Lovelace published a groundbreaking article on the use of venom sacs for the detection and treatment of HVA in 1956, the use of whole body extracts (WBEs) continued until the 1970s Floyd et al. (2). It was not until two case reports of hospitalized patients with recurrent allergic reactions treated with WBEs were published that the efficacy of WBEs was finally questioned Lichtenstein et al. (3) Busse et al. (4). This led to a key placebo-controlled trial published in 1978 that demonstrated that VIT was superior to WBEs in the treatment of HVA Hunt et al. (5). Following this and two other supportive trials, the U.S. Food and Drug Administration in 1979 approved venom extracts for the diagnosis and treatment of hymenoptera allergy. In addition, sting ants allergy is currently treated with Australian venom extracts [e.g., Jake Jump Ant Allergy (JJA)] and American WBEs [e.g., Imported Fire Ant Allergy (IFA)] Floyd et al. (2). With the in-depth understanding of VIT, people have made some attempts to explore the mechanism, diagnosis and treatment of allergic reactions caused by hymenopteran insects from the perspective of immune components. In view of the broad prospects of VIT in the allergic reactions of Hymenoptera insects and the existing knowledge gap, a comprehensive literature review is essential to identify emerging trends and research focuses, so as to guide further research in this area.

Bibliometric analysis is a method of quantitative investigation, examination and analysis of research results in specific fields. The main purpose of analyzing literature is to obtain detailed information such as authors, keywords, references, institutions, countries, etc. Bibliometrics provides a more objective and reliable analysis for research. Through structured analysis of a large amount of information, we can mine the topics of related research fields, identify changes in the direction of subject research, and discover future development trends. Systems scientometrics supported by computational and visual analytics provides an opportunity to improve the timeliness, accessibility, and repeatability of literature research in the research field. It not only provides a basis for researchers to analyze the hot spots and development trends of disciplines and predict the development direction of disciplines, but also provides a reference for discipline construction and talent training in hospitals and medical colleges. As far as we know, so far, there has been no bibliometric study devoted to the topic of hymenoptera VIT.

Our analysis mainly focuses on the cooperation between countries in the hymenopteran VIT, and the co-occurrence of keywords. We reviewed the literature before 2014, and there are few reports on Hymenoptera VIT. In the introduction, we clarified that the literature period from 2014 to 2024 was chosen to capture the most recent and relevant research in the field of venom immunotherapy of Hymenoptera allergy. This time frame was selected to reflect the current state of knowledge and emerging trends in this area. The main purposes of this study are: (1) to summarize the research of hymenoptera VIT from 2014 to 2024 under the background of globalization, (2) to discuss the popular research topics and their characteristics in this discipline, (3) to analyze the research directions with potential value based on the analysis of emerging trends.

## 2 Method and data

### 2.1 Data source

**Figure 1:**
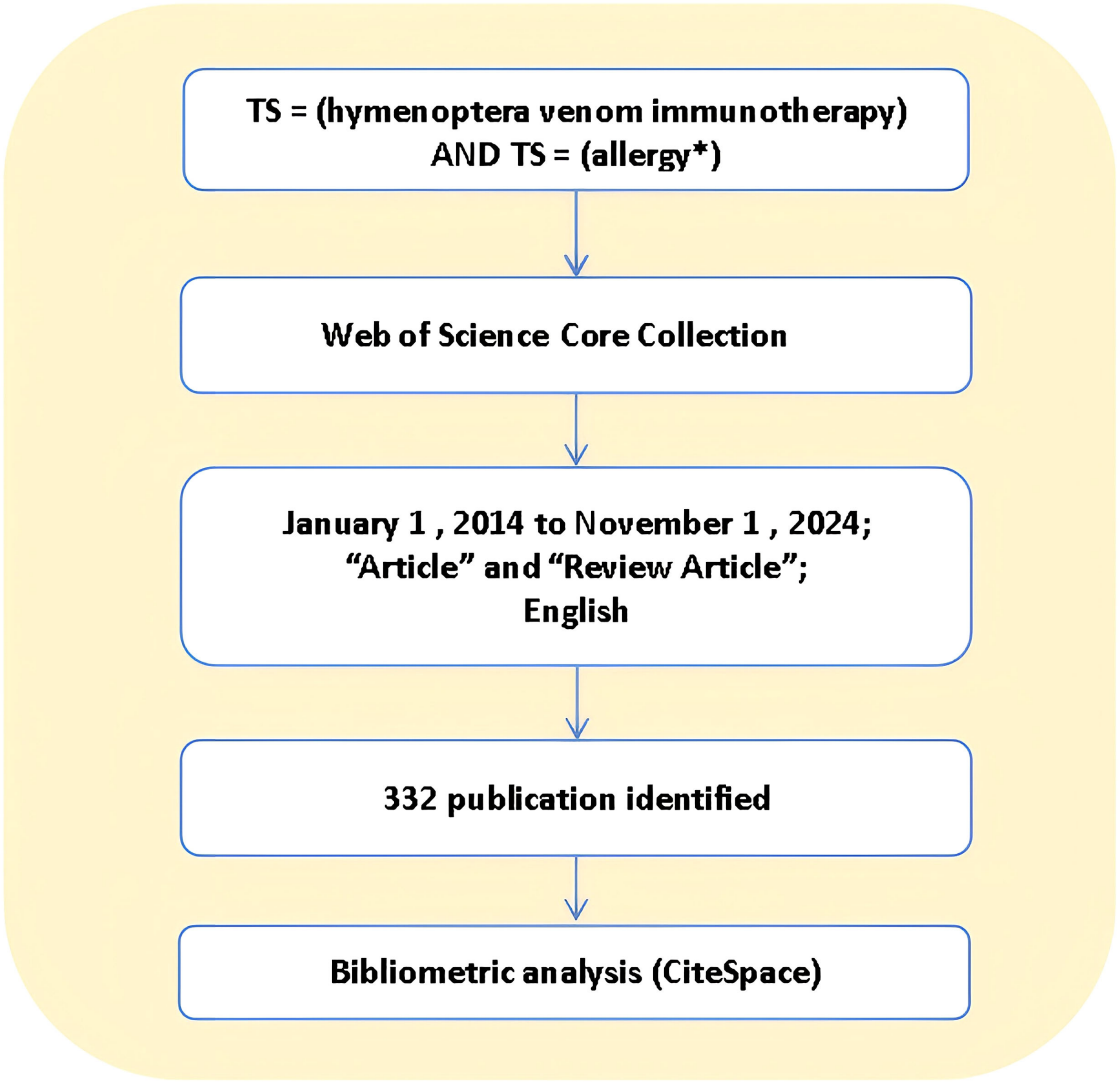
The flowchart illustrating the search strategy and analysis process for hymenoptera venom immunotherapy.

Web of Science is a comprehensive multidisciplinary core journal database. Search method: TS = “hymenoptera” AND “allergy *”, search time range: 2014-01-01 to 2024-11-01, search time is November 21, 2024. A total of 410 articles (259 articles, 120 reviews, 13 editorial materials, 10 meeting abstracts, 8 letters, 4 pre-published articles not included) were retrieved using this search term. A total of 351 articles were screened in English. Through the reading of the title, abstract and content of the article, the unrelated literature was further eliminated, and 332 articles were finally obtained, and 332 articles were analyzed by scientometrics. **Figure 1** shows.

### 2.2 Research methods

We export the retrieved articles in plain text format, including complete records and references, and then import them into CiteSpace 6.3.R1 for further analysis. When mapping visual knowledge numbers, we follow the main procedural steps of CiteSpace, including time slicing, thresholding, modeling, trimming, merging, and mapping. The core concepts of CiteSpace include burst detection, centrality, and heterogeneous networks, which can help to visualize research status, hotspots, and frontiers in a timely manner. Nodes in different maps represent authors, institutions, countries or keywords. The size of the node indicates the frequency of occurrence or reference, and the color of the node indicates the year of occurrence or reference. In addition, nodes with purple borders represent high betweenness centrality, and these nodes are usually identified as hotspots or turning points in a domain. Centrality is a key indicator for analyzing the importance of keywords. If the centrality exceeds 0.1, it indicates that the node is a central node, which is important and has great influence in the research. CiteSpace can also solve the problem of understanding the links or working relationships between documents, help users reduce cognitive gaps and identify key points and future trends in the research field. Therefore, in this study, the bibliometric method based on CiteSpace was used to analyze the existing papers related to the hymenoptera VIT. In addition, we also conducted a critical reading in order to conduct a more in-depth analysis of key research and provide key insights into the topic.

### 2.3 Data analysis

Using the analysis and retrieval results function of Web of Science, the retrieved documents were classified and stored. Excel was used to count the annual number of articles, countries, institutions and authors. At the same time, CiteSpace (6.3.R1) was used to extract important noun phrases from the title, abstract and keywords of the literature for literature co-word, co-occurrence and burst word analysis. In CiteSpace, the time span is selected from 2014 to 2024, the time node is set to 1 year, the node type is Country, Keyword, etc., the node strength defaults Cosine, the threshold value is TOP 50, and the network cutting function area is selected. ‘ Pathfinder, ‘ ‘ Pruning sliced networks ‘ and ‘ Pruning the merged network ‘ are selected for map analysis.

## 3 Results

### 3.1 Trend analysis of publication

**Figure 2:**
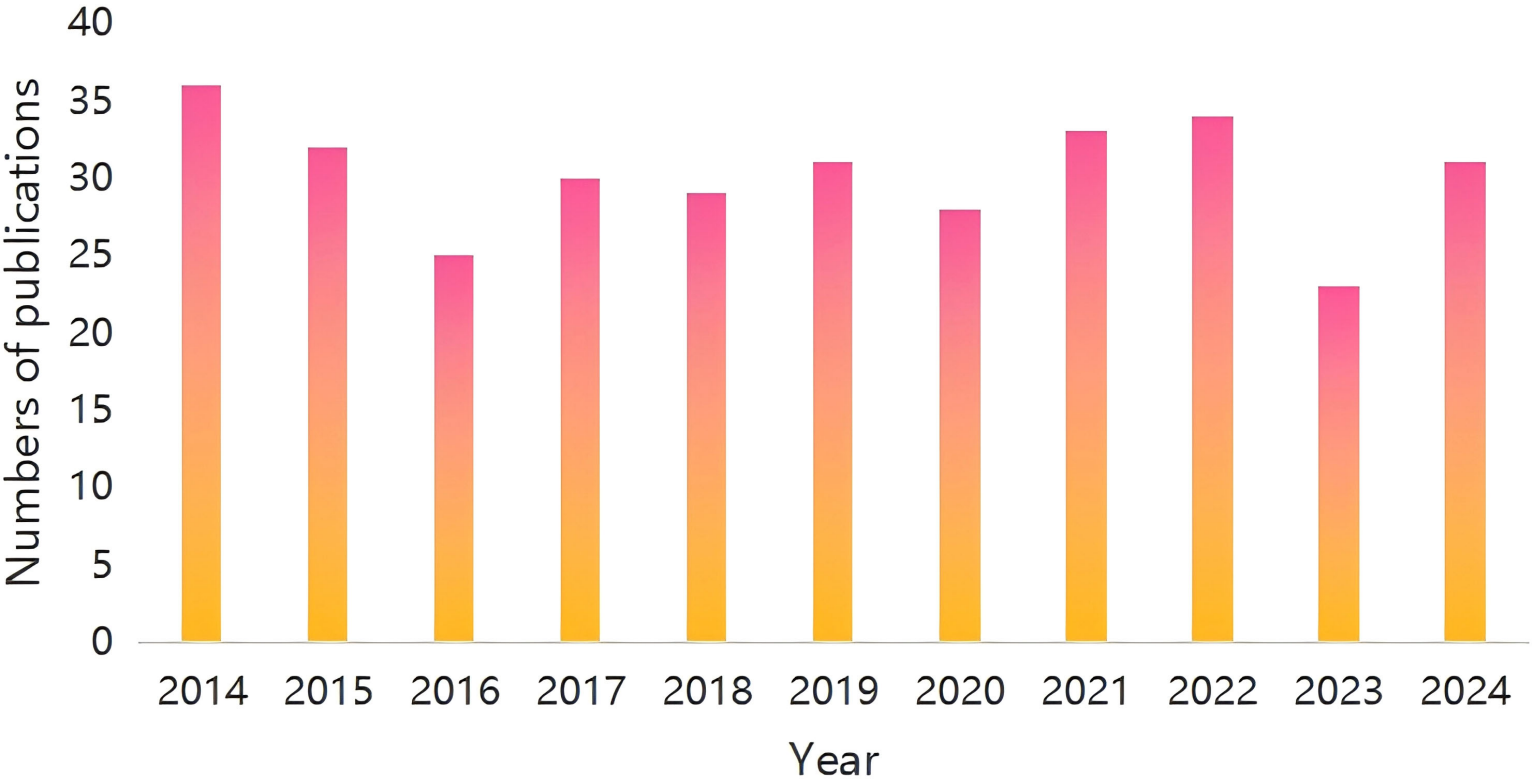
The time evolution of the total number of publications in the WOS database.

The trend of literature publication is an important indicator to measure the research and development of a certain field of the discipline. Therefore, drawing the distribution curve of the number of documents over time can effectively evaluate the research status of the discipline in this field and further predict its development trends. **Figure 2** shows the annual distribution of the research literature on the treatment of allergies caused by hymenoptera insects by venom immunotherapy on Web of science in the past 11 years. According to the calculation, the average annual number of published papers on Web of science in the past 11 years is only 33, and the annual number of published papers fluctuates between 25-36. The peak appeared in 2014, with 36 papers published, and 31 papers published in 2024. Considering the incomplete data in 2024, it is expected that the number of papers published in this year will exceed the previous records, which reflects the increasing interest and attention of the academic community in this field. Although the number of studies remains relatively low, the research work on the treatment of hymenoptera venom immunotherapy has developed rapidly. In the next few years, there will still be significant research opportunities and room for exploration within this domain.

### 3.2 Analysis of countries/regions and institutions

#### 3.2.1 Country/region analysis

**Figure 3:**
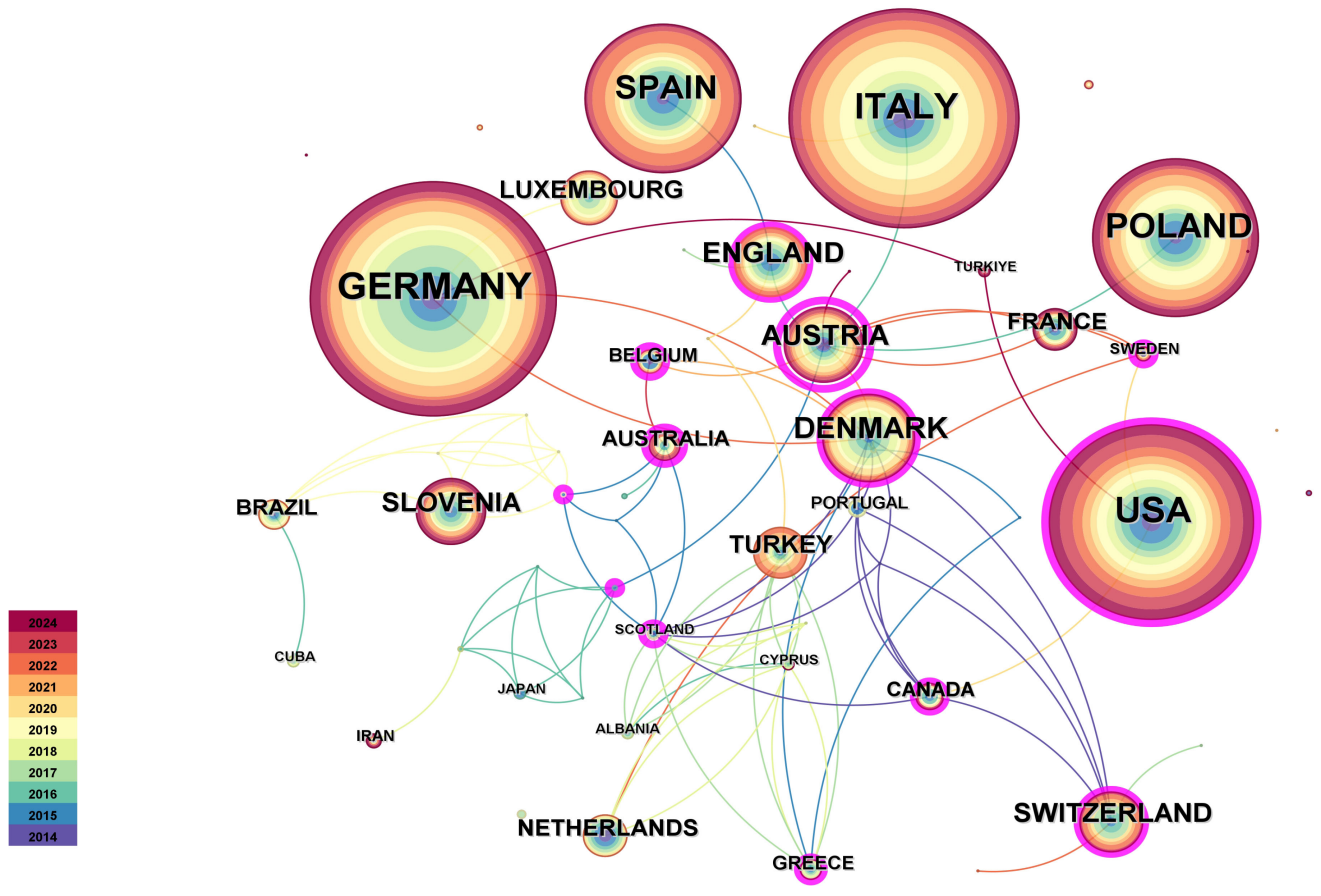
Cooperation map of countries/regions.

Through the quantitative analysis of the publishing countries/regions, we can not only identify the core countries/regions in the field of research on the immunotherapy of the allergic venom of hymenoptera, but also reflect the academic exchanges and cooperation among countries/regions in this field. Therefore, this study selects the country/region as the analysis object in CiteSpace, sets the Time Slicing as ‘ 20142024 ’, the Years Per Slice as 1, and the threshold as top 50, and finally obtains 55 network nodes and 317 connections. The national analysis map with a density of 0.0646. The thickness of the purple ring indicates the degree of the intermediate centrality. The centrality of the node in the network measures the importance of the node’s position in the network. **Figure 3** shows.

Frequency represents the number of occurrences of the country, and centrality represents the status of the country in this discipline. It is apparent that the descriptiveness of centrality reflects the connection of the country, that is, the more the connection of the country, the higher the centrality of the country, the richer the research of the country, and the more important the status of the country in this field.

Obviously, Germany is the country with the highest number of publications in this field, reaching 77, which is much larger than other countries. This shows that Germany is very concerned about the field of hymenoptera venom immunotherapy, and Germany is also developing rapidly in this field. In the leading state, the centrality of Germany is 0.08, which indicates that its transnational cooperation is relatively small. Secondly, Italy ranked second with a frequency of 72, and USA ranked third with a frequency of 64. From the perspective of centrality, the centrality of USA, ranked 3rd in the number of publications, is higher than that of Germany and Italy, which proves that USA has relatively more international cooperation. Poland and Spain have the worst centrality and almost no international cooperation. It can be seen from the year that the research of these 10 countries began in 2014, and the current frequency is high, which shows that these 10 countries have played a major role in the research field.

#### 3.2.2 Institutional analysis

**Figure 4:**
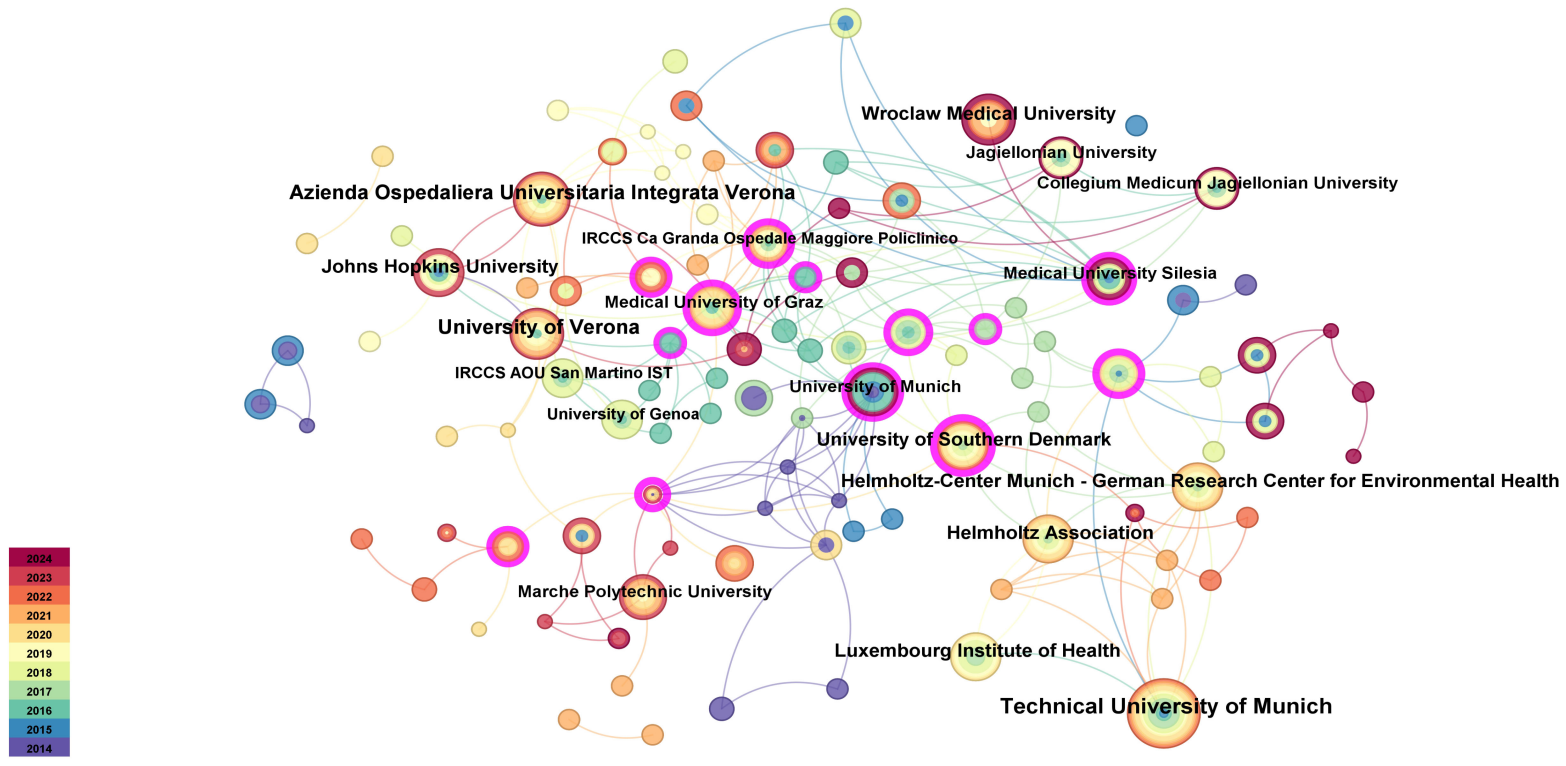
The institutional collaboration network.

Taking the institution in CiteSpace as the analysis object, the institutional analysis map of 115 network nodes and 317 connections with a density of 0.0331 was obtained (**Figure 4**). As shown in **Figure 4**, the nodes in the map are relatively dense, and the data are in the majority, with a total connection of 317, which indicates that institutional cooperation in the field of hymenoptera VIT research is relatively frequent. Most research institutions cooperate, and some of them have obvious regional characteristics. It is evident that continued cross-institutional research and collaboration are essential to furthering academic exchange in the field of hymenoptera VIT.

Hymenoptera VIT is also presented in **Table 1**. Technical University of Munich has the highest number of publications, reaching 23, ahead of other institutions, but its centrality ranking is relatively low, which indicates that Technical University of Munich has a high frequency of publications, but less cooperation with other institutions. Azienda Ospedaliera Universitaria Integrata Verona, University of Verona and Johns Hopkins University published earlier papers, indicating that they have made great contributions to the study of venom immunity in the allergic reaction of hymenoptera. At the same time, Azienda Ospedaliera Universitaria Integrata Verona also ranked second in the number of publications, with a frequency of 22, which indicates that it has attracted much attention in this research field, and the centrality is 0.06, at a upper level, showing that Azienda Ospedaliera Universitaria Integrata Verona has conducted in-depth research in this area in recent years, and pointed out the direction for the frontier research of hymenoptera VIT.

**Table 1:** Top 10 productive countries, institutions and authors.

Country	Count	Centrality	Institution	Author	Count	Centrality
Germany	77	0.08	Technical University of Munich	Golden, David B K	16	0.05
Italy	72	0.06	Azienda Ospedaliera Universitaria Integrata Verona	Nittner-marszalska, Marita	13	0.14
USA	64	0.21	University of Verona	Blank, Simon	12	0.01
Poland	52	0	Johns Hopkins University	Bonadonna, Patrizia	11	0.14
Spain	49	0	Wroclaw Medical University	Kosnik, Mitja	10	0.01
Denmank	29	0.11	Luxembourg Institute of Health	Passalacqua, Giovanni	9	0.02
Austria	25	0.72	University of Southern Denmark	Ollert, Markus	8	0.18
England	23	0.16	Helmholtz Association	Rueff, F	7	0
Slovenia	22	0.03	Helmholtz-Center Munich - German Research Center for Environmental Health	Cichocka-jarosz, Ewa	6	0.21
Switzerland	20	0.13	Jagiellonian University	Bilo, Maria Beatrice	6	0.32

#### 3.2.3 The co-citation analysis of authors and journals

Among the most frequently co-cited authors, Golden DBK ranks first, but his centrality is low, suggesting limited collaboration with other researchers. If he were to strengthen his collaborations, he could potentially bring even greater contributions to the medical community in the future. J Allergy Clin Immunol leads the citation frequency with 322 mentions, underscoring its authoritative role in the field of VIT and the significant attention its published articles receive within the academic community.

#### 3.2.4 The cluster analysis

**Figure 5:**
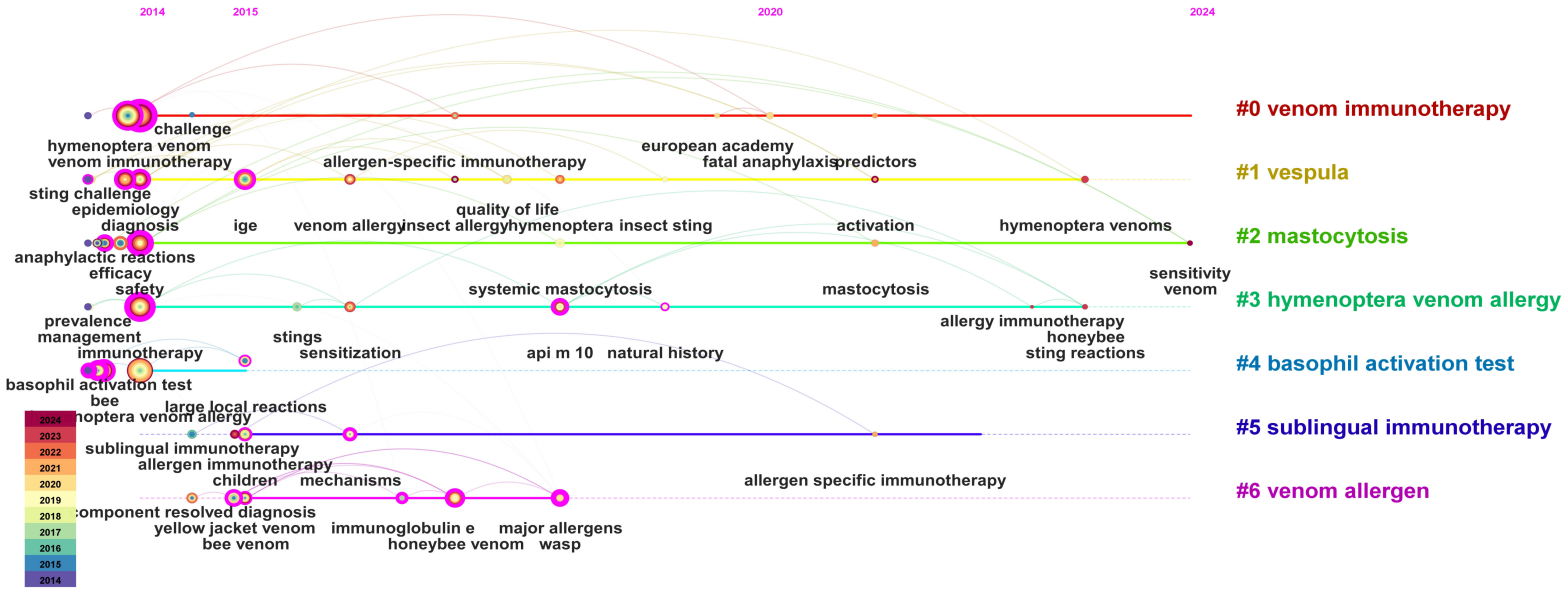
Research topics in the field of hymenoptera VIT in the past 11 years.

The keywords are clustered by CiteSpace, the cluster option is selected, and the “Pathfinder “, “Pruning slicednetworks” and “Pruning the merged network “ algorithms are used to cut the connection lines to ensure the rationality of clustering classification. The results are shown in **Figure 5**, reflecting the research topics of monitoring in the field of hymenoptera VIT in the past 11 years. The cluster number is the topic obtained by clustering the keywords by LLR algorithm. A total of 7 clusters are obtained, and the information of each cluster is shown in the figure.

In this study, a total of 7 research topics were generated,namely,#0 venom immunotherapy;#1 vespula;#2 mastocytosis;#3 hymenoptera venom allergy;#4 basophil activation test;#5 subling immunotherapy;#6 venom allergen. Different clusters cover different keywords, Theme#0 and#5 focus on the diagnosis of Hymenoptera venom allergy;Themes#1 and#3 address hymenoptera VIT; Theme#2 examines the safety and efficacy of immunotherapy using hypersensitive venom from Hymenoptera insects; Theme#4 emphasizes the monitoring of indicators related to the diagnosis of allergic reactions to Hymenoptera venom; Theme#5 explores the mechanisms of immunotherapy with hypersensitive venom from Hymenoptera insects and its application in pediatric populations; and Theme#6 investigates allergens responsible for allergic reactions to the venoms of different Hymenoptera species (such as yellow jackets, wasps, and bees). The research year of cluster#2 is concentrated in 2014, indicating that the research topic of cluster#2 is relatively traditional. The year of cluster#3 is more concentrated in 2022 and 2023, which indicates that the research topic of cluster#3 is closer to the current research trend.

#### 3.2.5 Research frontier and trend analysis

**Figure 6:**
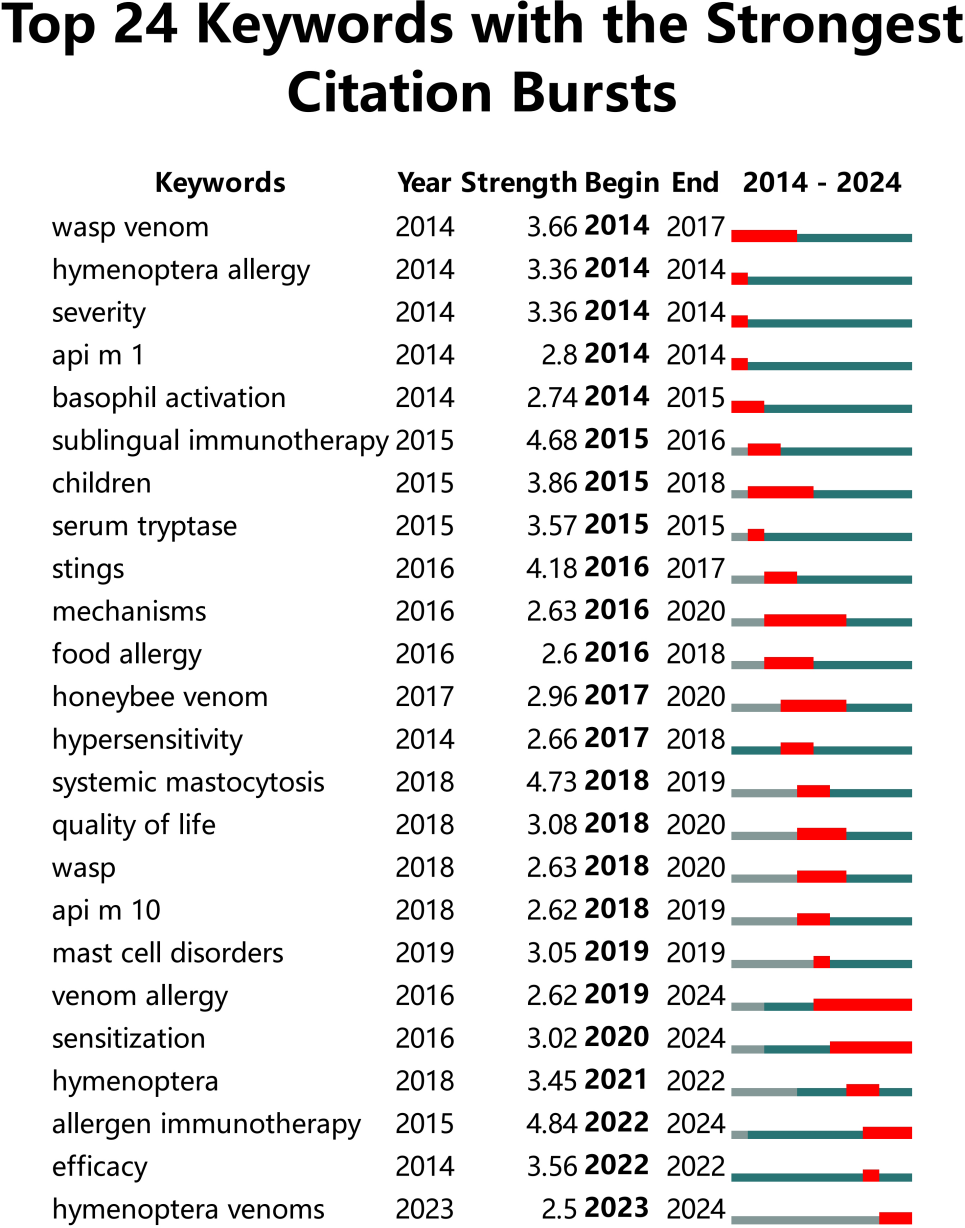
Bursts of keywords.

In this investigation, the bursts detection algorithm of CiteSpace software was used to obtain the evolution map of keyword hotspots in the field of hymenoptera venom immunotherapy on Web of science, that is, keyword emergence, as shown in **Figure 6**. This investigation generated a total of 24 keywords in the top 24 of the research field, and the specific emergence intensity and hot spot duration are shown in the table. The time interval of the emergence is indicated by the green line. It is found that the burst time period of a topic category is shown as a red line segment, indicating the start year and end year of the burst duration.

It can be observed from **Figure 6** that the prominent research trends in the immunotherapy of hymenoptera venom allergy persisted from 2014 to 2024. In 2014, the primary research focus at this stage were wasp venom, hymenoptera allergy, severity, api m 1 and basophil activation. Over time, 2018 became a node, and new topics and hot spots began to emerge, such as systemic mastocytosis, quality of life, wasp, and api m 10. This shows that mastocytosis, the quality of life of people receiving venom immunotherapy, wasps, and allergens similar to api m 10 that may have diagnostic value for allergic reactions of hymenopteran insects are frontier topics in the current field. Looking ahead, researchers may be committed to the implementation and promotion of more accurate, reliable, safe and effective individualized hymenoptera venom immunotherapy.

## 4 Discussion

In this study, we conducted a comprehensive scientometric study on the development of hymenoptera VIT in the past 11 years. We examined the characteristics of the use of hymenopteran VIT from multiple perspectives: the characteristics of publication output, cooperation between countries, and co-occurrence analysis of subject categories and keywords. The attention of this research field is not evenly distributed around the world, but mainly concentrated in Europe and North America. At the same time, international cooperation between Europe and North America is relatively close. Technical University of Munich, Azienda Ospedaliera Universitaria Integrata Verona, University of Verona and other institutions are actively exploring and publishing papers in the research field.

In the diagnosis of hymenopteran insect allergy, serum IgE (sIgE) detection is widely recognized as an important means to diagnose venom allergy because of its high sensitivity in detecting allergen-specific IgE Köhler et al. (6). However, its diagnostic accuracy remains controversial due to the possibility of false positives and false negatives. Species-specific differences may also affect the diagnostic utility of sIgE detection. For example, individuals allergic to wasp venom and individuals allergic to bee venom may exhibit different sIgE profiles, which makes diagnosis and treatment more complicated. In the absence of clear sensitization to insects, the double positivity of bee venom and wasp venom requires further laboratory tests, such as IgE inhibition test or basophil activation test (BAT). These tests are often expensive, time-consuming and difficult to interpret, which limits their clinical application Eberlein et al. (7).

The main allergens associated with bee venom include phospholipase A2 (Api m 1), hyaluronidase (Api m 2), and various basic toxin peptides (Api m 4), all of which are medium and high abundance proteins. Other low-abundance allergens have also been cloned and expressed, such as Api m 10 and Api m 12 Vega-Castro et al. (8). Studies have evaluated the sensitization spectrum of a series of recombinant allergens in patients with bee venom allergy. These recombinant allergens do not contain carbohydrate cross-reactive epitopes (CCDs) Eberlein et al. (7), emphasizing the advantages of component-based diagnostic methods in improving sensitivity and revealing different sensitization patterns. Studies have shown that antigen 5 is not a suitable marker for distinguishing between wasp toxin allergies. However, studies have found that the combination of sIgE measurement and basophil activation test (BAT) is expected to be used as a strategy for the diagnosis of clinically relevant sensitization of wasp toxins Schiener et al. (9).

VIT can regulate the innate immune system, thereby inhibiting allergic immune response Dreschler et al. (10). Within a few hours after VIT administration, basophil activation inhibition mediated by histamine type 2 receptor was observed Novak et al. (11). Subsequently, VIT inhibited IgE production and induced the production of specific IgG4 antibodies Akdis et al. (12) van de Veen et al. (13), thereby promoting peripheral tolerance. One of the characteristics of VIT is the initial increase in toxin-specific IgE (sIgE) levels, followed by a decrease over time, while toxin-specific IgG and IgG4 antibody levels increase Varga et al. (14) Kemeny et al. (15) Müller et al. (16). These antibodies play a crucial role in the efficacy of VIT. Although a large number of studies have been conducted in the field of insect sting allergy, the exact mechanism of VIT is still not fully understood.

In the past, significant progress has been made in the study of VIT for the sting allergy of hymenopteran insects, mainly focusing on the mechanism of the response of specific IgE and IgG4 antibodies to toxin components Sturm et al. (17), highlighting their key role in immune regulation and tolerance induction. Studies have shown that VIT can effectively reduce the incidence of severe allergic reactions, and the emphasis on individualized treatment is increasing. Future research should prioritize the optimization of treatment options and enhance patient compliance to improve clinical outcomes Floyd et al. (2). VIT is currently the only treatment that can potentially prevent further systemic sting reactions. It is reported that the effective rate of VIT in the treatment of bee stings is 77-84%, the effective rate of VIT in the treatment of wasp stings is 91-96%, and the effective rate of VIT in the treatment of ants stings is 97-98% Müller et al. (18) Ruëff et al. (19) Brown et al. (20) Tankersley et al. (21). Subcutaneous VIT is currently the most effective way of administration and has a lasting effect on most patients Bilò et al. (22).

Asthma is considered to be a potential risk factor for severe VIT response and may even be the main cause of death, especially when asthma is poorly controlled Epstein and Calabria (23). The presence of cardiovascular diseases (such as hypertension or arrhythmia) may complicate the safety of VIT. Some drugs, such as *β*-blockers, may increase the risk of severe cardiovascular events during allergic reactions and may interfere with the response of adrenaline. Although many studies have shown that VIT is still safe and effective for such patients under careful management, and cardiovascular disease itself is not a contraindication to VIT Pitsios et al. (24), the field is still complex and requires more detailed research. Personalized VIT programs for comorbidities such as asthma and cardiovascular disease are key to ensuring patient safety and successful treatment.

The results have two limitations. First of all, due to the relatively small amount of literature available in this field, the results of bibliometric analysis are limited and may not fully describe all aspects of the research field. In addition, due to the design of the software, it is difficult to represent the newly published high-level literature in the visual analysis results compared with previous studies. In the future, we will continue to improve these defects to improve the accuracy of trend prediction. Through the above analysis, we get the following key conclusions: hymenoptera VIT has become a global participation and cooperation research topic, which requires more researchers to participate, especially a large number of randomized controlled trials. In summary, this study used CiteSpace software to visualize the keywords of countries and institutions in the field of hymenopteran VIT research in WOSCC from 2014 to 2024. As far as we know, there are limited studies on bibliometric analysis and visualization of monitoring technology in the field of hymenoptera VIT resuscitation using CiteSpace. This study is a meaningful attempt in this direction.
